# Research and development of fully enclosed wire-shell support structure technology for deep soft rock roadway based on TRIZ theory

**DOI:** 10.1038/s41598-024-53972-7

**Published:** 2024-02-08

**Authors:** Weijing Yao, Chengjun Wang, Jianyong Pang, Yushan Liu, Jinsong Zhang

**Affiliations:** 1 Base for innovative Methods Promotion Application and Demonstration of Anhui Province, Anhui University of Science and Technology, Huainan, 232001 Anhui Province China; 2 School of Civil Engineering and Architecture, Anhui University of Science and Technology, Huainan, 232001 Anhui Province China; 3 Anhui Key Laboratory of Mining Construction Engineering, Anhui University of Science and Technology, Huainan, 232001 Anhui Province China

**Keywords:** Energy science and technology, Engineering

## Abstract

The TRIZ theory was used to accurately discover the problems to be solved in the design of roadway surrounding rock control technology. This paper tried to solve the complex issue of surrounding rock control in deep roadways from a new perspective. Based on the functional component analysis and causal axis analysis of the problem’s primary reason, simultaneously, the surrounding rock control technology was optimized through technical contradiction analysis, physical contradiction analysis, and substance and field model analysis. As a result, a fully enclosed wire-shell support technology was proposed. Finally, taking the typical soft rock roadway engineering of Pansan Coal Mine in Huainan Mining Area, Anhui Province, China, as the engineering background, the engineering application and effect evaluation were completed. This paper provides a reference for controlling the instability of deep soft rock roadways in coal mines. A new idea of optimizing roadway support engineering based on TRIZ theory was proposed.

## Introduction

Genrich Altshuller established TRIZ theory from the Soviet Union, which means the Theory of Invention Problem Solving. TRIZ is a systematic process for solving a particular problem or conflict of innovative methods. Adopting this theory can help designers systematically analyze problems, quickly find the nature of issues, accurately discover the problems to be solved in design, find innovative solutions, shorten the invention cycle, and improve the success rate of the invention^1,2^. After being introduced into China, TRIZ theory has been widely applied in the military, machinery, chemical industry, information technology, management, civil engineering, medical treatment, patent layout, and many other disciplines after a period of development and promotion^3–7^. It has been proved that TRIZ theory helps accelerate the process of invention and creation, and high-quality, innovative products can be obtained, which is the most effective way to realize innovative design and conceptual design^8,9^.

As the primary energy source in China, coal has significantly contributed to the rapid development of the Chinese industry. Deep mining has become a usual trend with the depletion of external resources and the gradual increase in energy demand. It means that future excavations will be carried out in an environment with high in-situ stress and poor surrounding rock conditions. Chinese coal mines’ new digging roadway lengths are above 12,000 km yearly^10^. A large number of roadways are excavated in the soft rock. With the increasing mining depth, it can be challenging to maintain the stability of soft rock roadways. Therefore, roadway support theory and technology are the core of coal mine rock control research^11^. According to the geological and production conditions of coal mine roadway, the deformation and failure characteristics of the surrounding rock, China has developed various forms of roadway support technology, such as metal support, shotcrete support, net anchor shotcrete, and enhanced metal support, high strength arc-bearing plate, anchor cable support in recent years. However, shotcrete support, shotcrete mesh support, and U-shaped steel retractable metal support were widely used in China^12–15^. According to the principle of support and reinforcement, it can be divided into three forms. ① Active support method used in surrounding rock; ② Passive support method acted on roadway surface; ③ Combined support suitable for complex roadway^10^. Currently, the mine roadway support design is still based on the analogy method of engineering experience, and the guidance of scientific methods to solve engineering problems needs to be improved. The geological conditions of coal mines in China are complex. There are many challenging roadways, for example, ultra-deep roadways with very soft and broken surrounding rock^16^, strong mining response and rock burst^17^, and vigorous floor heave^18^. Therefore, using scientific methods to find practical technology to effectively control large deformations on roadways is of great significance to ensure the safety and efficient production of coal mines. However, TRIZ theory is an efficient scientific method to explore this problem. Some engineers and scholars also developed new roadway support technology through TRIZ theory. For example, in a thick and hard roof that is extremely hard to break and cave, a long cantilever extrudes the roadway and casing deformation and failure of surrounding rock. Based on TRIZ theory, Yang et al.^19^ proposed the combined technology of softened gob roof comprising directional cumulative blasting and non-directional reinforced blasting, which has good engineering effectiveness. He et al.^20^ carried out a structural innovation design of an entire section anchor laying support robot with parallel operations such as walking, laying nets, supporting, anchoring, and drilling. Jia et al.^21^ designed a new energy storage function for yielding anchor bolts. He et al.^22^ developed a special transporter and transportation technology for heavy hydraulic support shaft transportation, providing efficient and safe trackless transportation technology. This shows that engineers and scholars in underground support in coal mines have paid TRIZ theory more and more attention.

The above research on improving roadway surrounding rock control has achieved good application. This paper addresses the problems of large deformation, fast speed, and complex control of soft rock roadways. The TRIZ theory of functional component analysis, causal axis analysis, substance and field model analysis, and technical contradiction analysis were completed. Inspired by the TRIZ invention principle of flexible shell and pre-action, a fully enclosed wire-shell support technology was proposed. Finally, the roadway restoration engineering was completed in the Pansan Coal Mine, Anhui Province, China. The application of TRIZ theory in roadway support design of coal mines is widened, which provides a reference for related engineering.

## Research methods and framework

For the problem of soft rock roadway support, the method combining theoretical analysis and typical engineering application is adopted. The specific steps are divided into three stages.

1. The first stage is system analysis, mainly using system completeness development and causality analysis. First, according to the development of system completeness, the essential system components must have been analyzed, and the relationship between the components is determined.
2. The secondary stage is problem definition. In this stage, technical contradiction analysis, physical contradiction analysis, and substance and field model analysis are used. A fully enclosed wire-shell support technology was proposed based on the 40 invention principles of TRIZ theory.
3. The third stage is problem-solving. In this stage, the reasonable feasibility of the proposed new support is determined through the combination of laboratory tests, numerical calculations, and engineering application.

**Figure 1:**
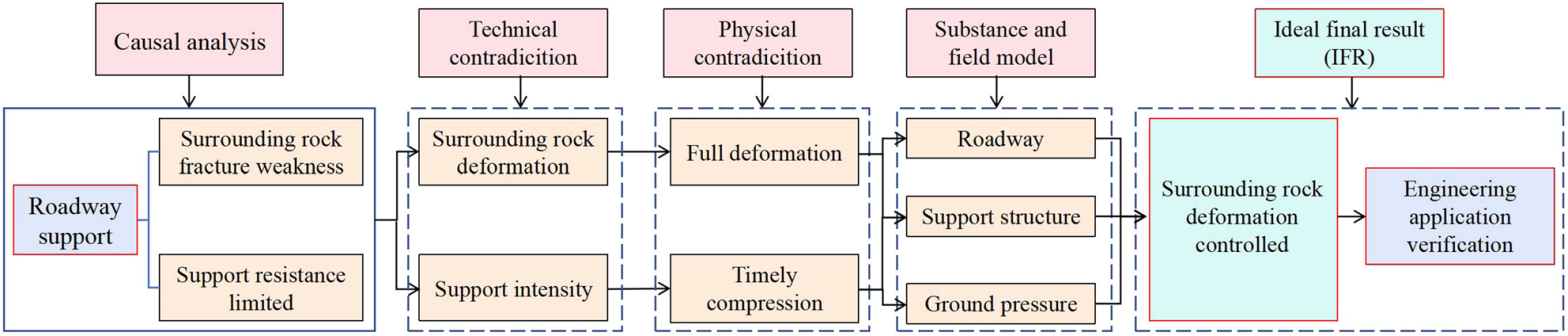
Theoretical framework for solving the roadway support.

The theoretical framework for solving the roadway support problem in this paper is shown in Fig. 1.

## System analysis of roadway surrounding rock control technology

### Problem description

**Figure 2:**
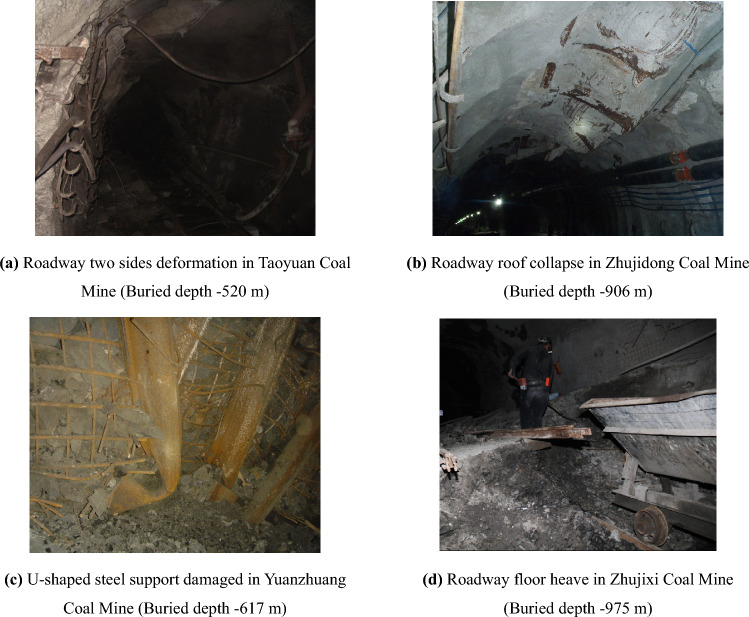
Typical failure of soft rock roadway support structure in Huainan and Huaibei Coal Mine Area of China.

Coal energy supported the rapid and stable development of China’s economy for a long time. With the normalization of deep mining, a deep-buried roadway with high stress becomes increasingly common^23,24^. Nowadays, significant deformation of roadway surrounding rock occurs in practical engineering. Figure 2 shows typical roadway deformation and failure in Huainan and Huaibei Coal Mine Area, Anhui Province, China, including two sides deformation (Fig. 2a,b), roof collapse (Fig. 2c), and floor heave (Fig. 2d).

In China, anchor mesh shotcrete support is often used in roadways with little surrounding rock pressure. U-shaped steel retractable support is widely used in roadways with more significant surrounding rock pressure^25^. However, the reduction of U-shaped steel support is less than 20% of the roadway section, which cannot effectively control roadway deformation. In addition, sizeable lateral pressure and uneven distribution of surrounding rock pressure make the support lose stability and shrinkage, weakening the superior mechanical properties of the support^26^. In a word, the stability control of roadway surrounding rock is a contradictory technical system of surrounding rock deformation and support resistance. It is urgent to use lightweight support. The strength is higher than that of anchor mesh shotcrete support, and the cost is lower than that of U-shaped steel support.

### Functional component analysis

From the perspective of TRIZ theory, the functional component analysis shows that the technical system of the engineering problem is roadway support measures, and the primary function is to maintain the stability of the roadway surrounding rock^27,28^. System components include rock bolts, anchor cable, steel mesh reinforcement, U-shaped steel support, shotcrete, grouting slurry, etc. The corresponding supersystem components and subsystem components are shown in Table 1.

**Table 1:** Functional component analysis.

Supsystem component	System component	Subsystem component
Roof of roadway	Rock bolt	Tray, bolt body, binder
Side of roadway	Anchor cable	Tray, cable body, binder
Floor of roadway	Steel mesh reinforcement	Longitudinal reinforcement, transverse reinforcement
Electromechanical equipment	U-shaped steel support	Single U-shaped steel arch, connector
Mine worker	Shotcrete	Cement, pebble, sand, additive
	Grouting slurry	Cement, sand, sodium silicate

**Figure 3:**
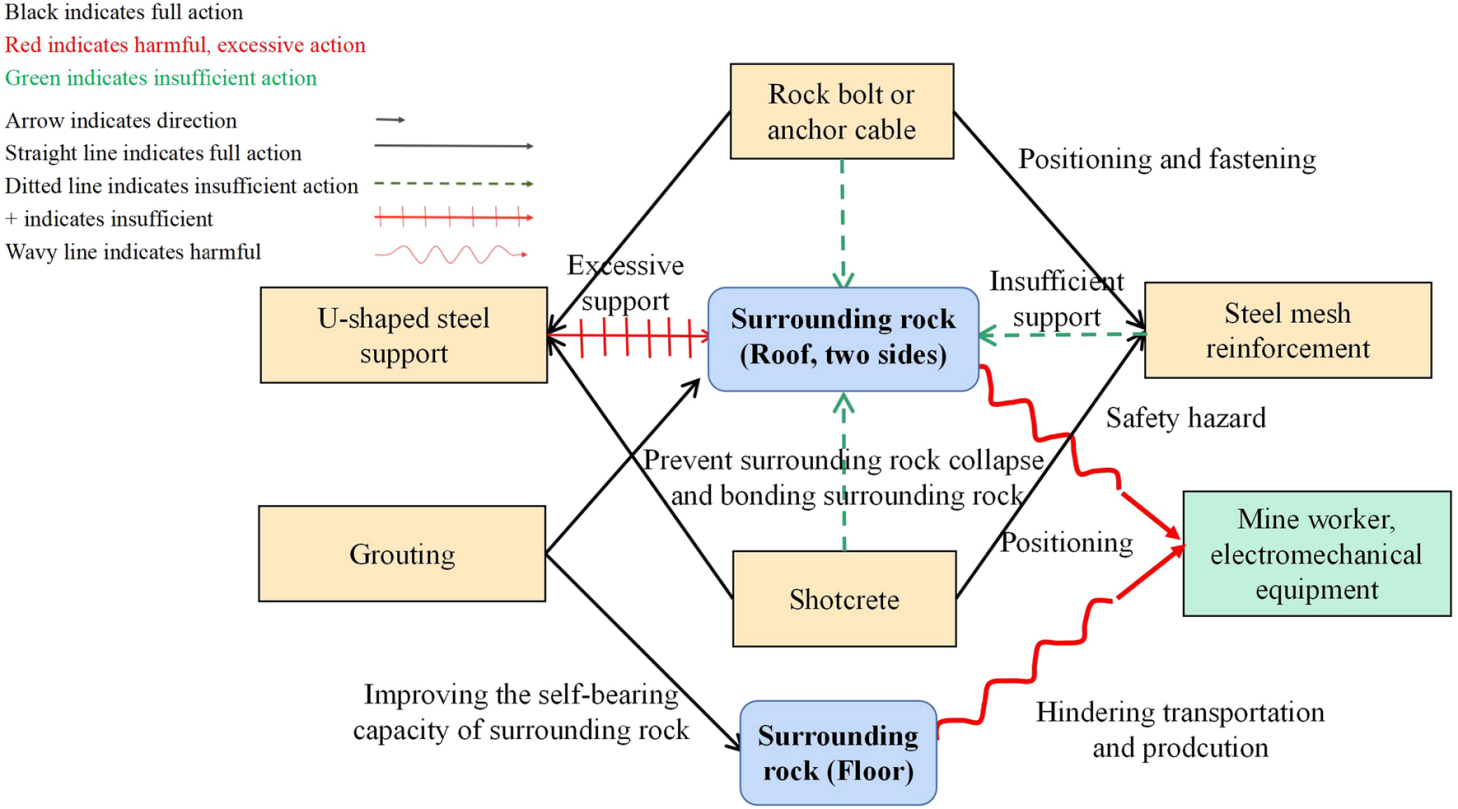
Functional component model.

Based on this analysis, the functional component model was drawn in Fig. 3. As can be seen from figure, there are two commonly used support measures: steel mesh reinforcement and U-shaped steel support. They are taken as the core of the support system. Other support measures benefit positioning, fastening, raising, strengthening, etc. In addition, further support measures prevent the surrounding rock’s collapse and improve the surrounding rock’s self-bearing capacity, which plays an auxiliary function. It shows that the roof and two sides of the surrounding rock harm personnel safety, while the floor of the surrounding rock hinders transportation and production. The analysis shows that the deformation and failure of the surrounding rock is the less support resistance to support the rock mass. However, the U-shaped steel support is excessive and needs a better economy. Moreover, the floor is neglected and lacks engineering support due to fewer personnel and mechanical equipment damage.

### Causal axis analysis

**Figure 4:**
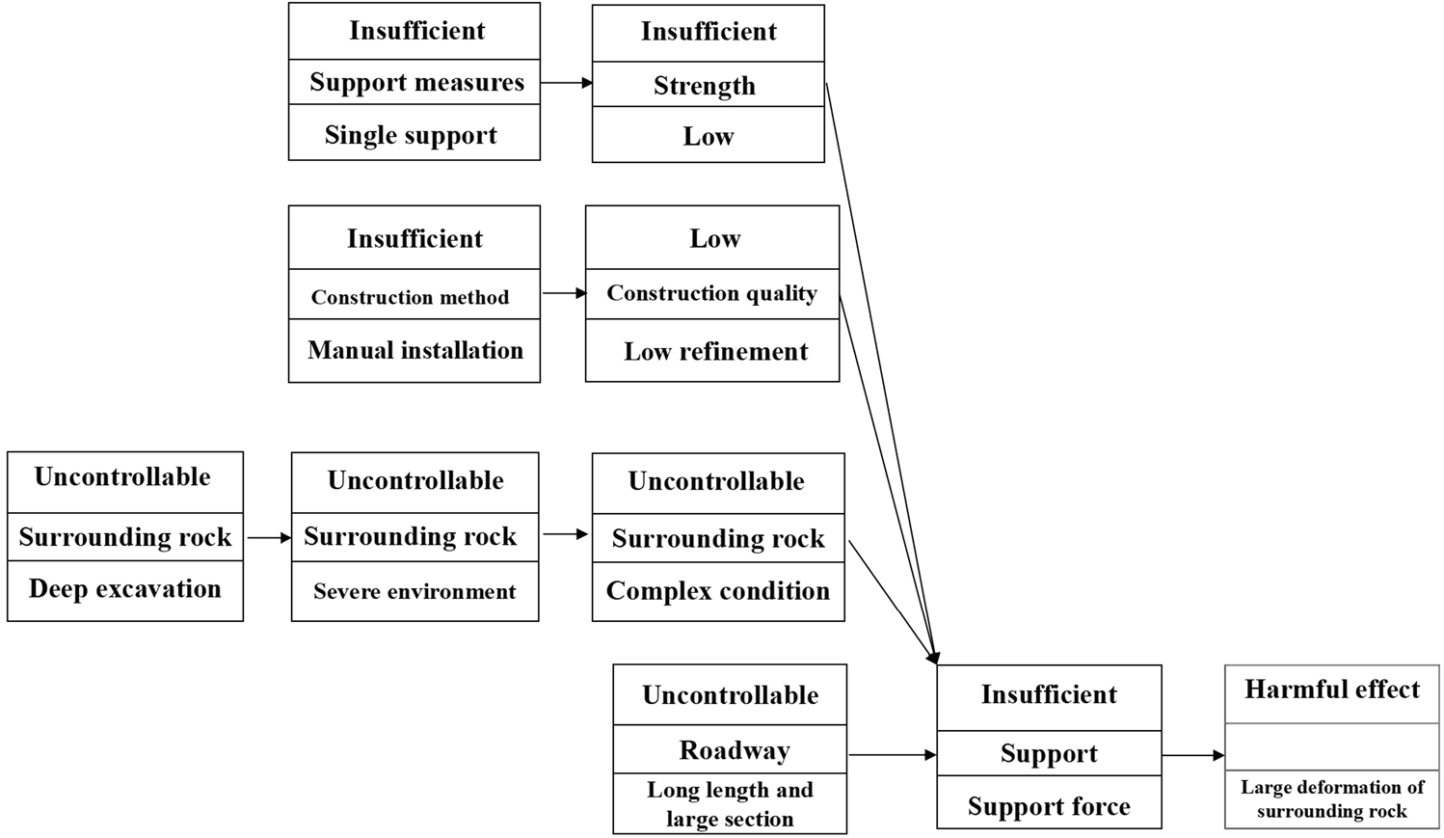
Causal axis analysis.

The causal axis analysis is shown in Fig. 4. As seen in figure, the main reason for the large deformation of the roadway surrounding rock is the insufficient supporting force of the supportive measures. Moreover, the roadway section continues to expand, the roadway length continues to extend, and the complex environment of surrounding rock is uncontrollable. Support strength, construction quality, and construction method are the complex causes of insufficient support strength.

## Optimal conceptual design of roadway surrounding rock control technology

### Technical contradiction analysis

Through the above functional components and causal axis analysis, the reason for the problem is the contradiction between the economic and adequate support and the large deformation of the roadway surrounding rock. Although the financial cost of anchor mesh shotcrete support is low, it has low strength and can only prevent stone collapse. However, the price of U-shaped steel support is relatively high, and the shrinkage of U-shaped steel cannot meet the requirements when the surrounding rock deformation is large. In addition, the floor heave is generally significant due to the unsupported floor.

The TRIZ invention principle was used. The strength and shape were a contradiction. The improvement parameter is strength, and the deterioration parameter is shape. The invention principles are 10, 30, 35, and 40, and the specific meanings and direction of the innovation idea are shown in Table 2^29^.

**Table 2:** Principle of technical conflict resolution.

Serial number	Name	Innovative direction
10	Pre-action	① Apply the necessary changes to the object (in whole or at least in part) in advance
		② Preposition object so that it begins to function in the most convenient location without wasting transport time
30	Flexible shell or membrane	① Flexible shells or membranes are used instead of conventional structures
		② Flexible shells or membranes are used to isolate from the external environment
35	Physical/chemical state change	① Change aggregation state (matter state)
		② Change the concentration or density
		③ Change flexibility
		④ Change the temperature
40	Composite material	Composite materials are used instead of homogeneous materials

**Figure 5:**
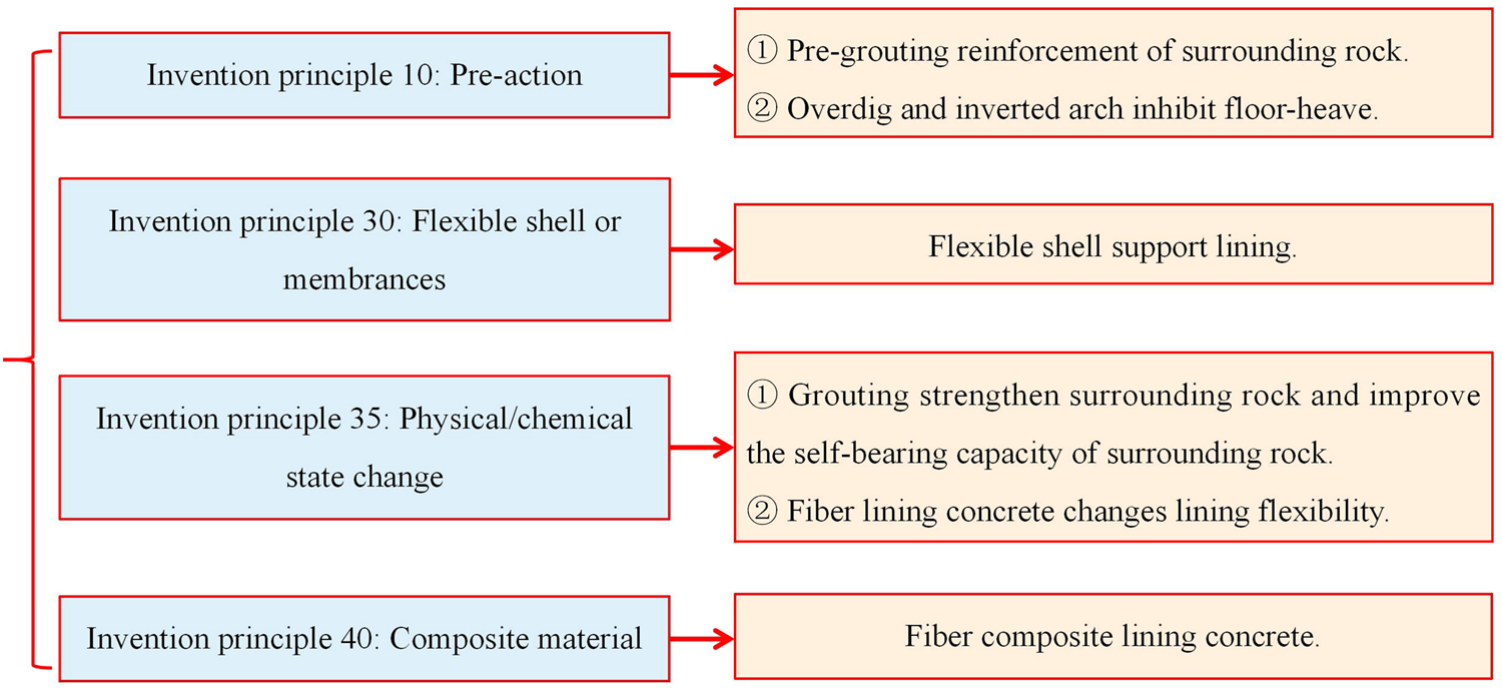
Conceptual design ideas from invention principle.

Through the analysis and careful consideration of the creative idea direction provided by the above invention principle, some preliminary conceptual ideas have been put forward, as shown in Fig. 5, and the detailed explanations are as follows.

1. Based on invention principle 10, the principle of pre-action provides to impose the necessary modifications on the object. For this research field, the roadway surrounding rock can be strengthened by grouting materials, and the broken rock mass around the soft rock roadway can be changed into a complete rock mass in advance to improve the self-bearing capacity. For the roadway floor heave, the part of the floor heave can be overdue to complete the pre-action, and the roadway floor can be made into an inverted arch shape.
2. Based on invention principle 30, a flexible shell or thin film provides a flexible surface or delicate film structure instead of a conventional system. The roadway support lining structure can be designed as a flexible shell to replace the traditional anchor mesh spray or U-shaped steel support. For example, the steel mesh shell structure used in airports and railway station ground buildings can enhance the deformation resistance.
3. Based on invention principle 35, the principle of physical or chemical state change provides for changing a physical state. Similarly, grouting materials can be injected into the roadway surrounding rock to change the physical condition of the broken surrounding stone, which can improve the self-bearing capacity of the surrounding rock.
4. Based on invention principles 40, composite materials, and 35, physical or chemical state change provides for changing compliance. Composite materials can be used instead of homogeneous materials. Composite materials can change shotcrete’s deformation and failure characteristics from rigid to flexible. For example, reinforced material with solid tensile properties, such as fiber mixed with shotcrete, can meet the strength requirements and have a specific deformation resistance.

### Physical contradiction analysis

**Figure 6:**
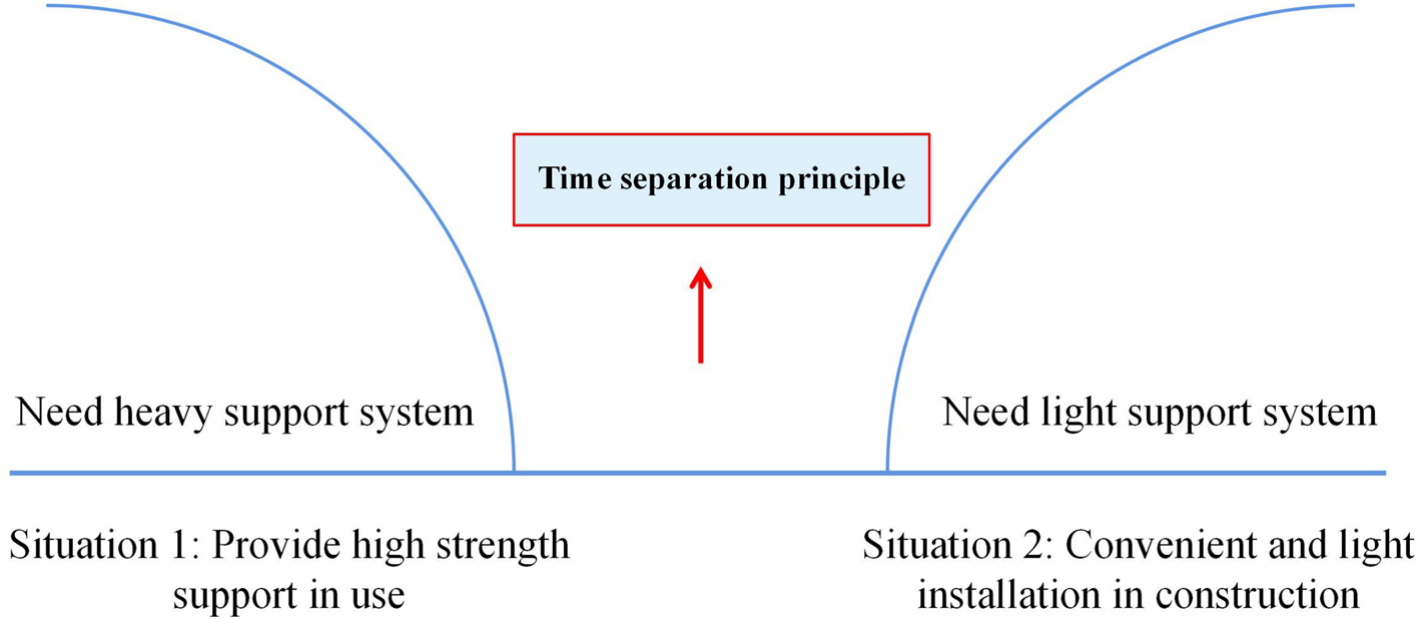
Design ideas from separation contradictory demand.

From the system functional analysis, providing high support strength after the completed support is desirable, thus requiring a heavy-duty support system. However, it is hoped that installing and reducing labor intensity will be accessible in supporting construction. Therefore, it needs a lightweight support system. As shown in Fig. 6, this type of problem can be solved using the principle of temporal separation in the separation contradiction.

Prefabricated and mechanical construction methods were used to improve the quality and accuracy of the support system, which can improve strength and reduce labor intensity and insufficient power caused by low construction accuracy.

### Substance and field model analysis

**Figure 7:**
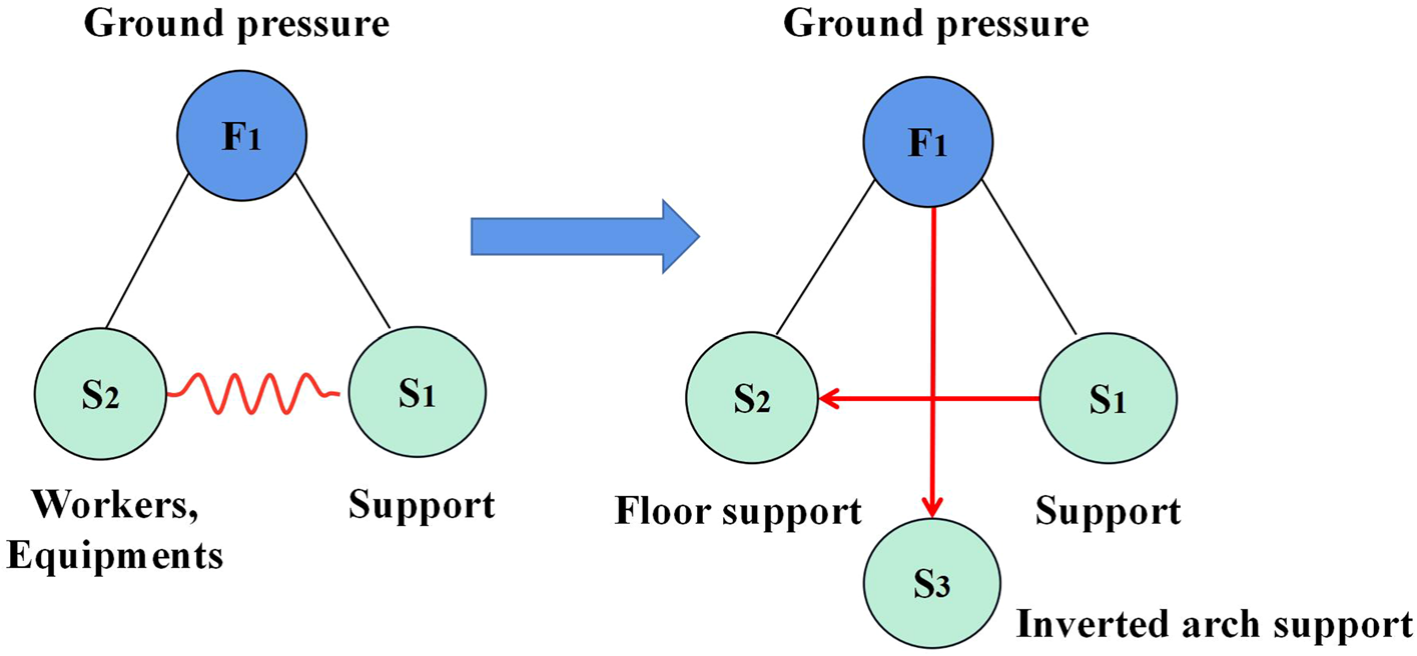
Substance and field model analysis.

According to the system function analysis and causal axis analysis, the roadway floor is often neglected to support, which results in severe floor heave, hindering transportation and production. Therefore, general solution 2 was used to solve it. A third substance was added to this solution to prevent the production of harmful substances, as shown in Fig. 7.

According to Fig. 7, adding inverted arch support was adopted to prevent the harm of floor heave, and the design idea of inverted arch support to contain floor deformation was generated.

## Innovative scheme: a fully enclosed wire-shell support structure technology

### Innovative scheme

According to the practice of roadway surrounding rock control engineering to optimize the above conceptual design, inspired by the invention principle 35 flexible shells and the invention principle 10 pre-action, drawing on the advantages of lightweight and robust stability of the flexible structure of the ground reinforced shell, a fully enclosed wire-shell support structure technology has been developed by our research group, Anhui University of Science and Technology, China. This technology has been declared a Chinese invention patent. The patent number is 201510909386.2^30^.

**Figure 8:**
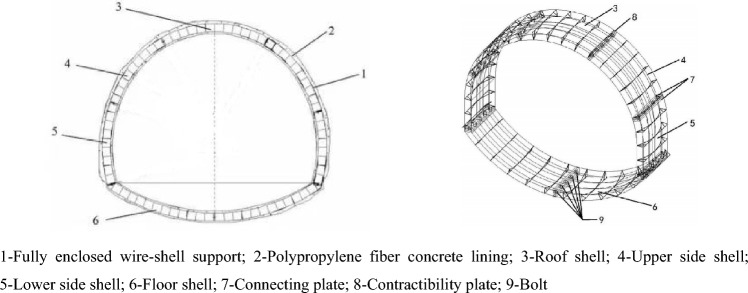
Fully enclosed wire-shell support structure^30^.

A schematic of this support structure is shown in Fig. 8. It can be seen from the figure that the structure is a shell structure welded with steel on the ground. The outer surface consists of a steel mesh layer, which can support the surrounding rock surface, and the internal three-dimensional steel mesh keeps the outer steel mesh. In addition, the design of the fully enclosed structure is also in line with the idea from substance and field model analysis, which can contain the deformation of the roadway floor by inverted arch support. For this new support structure, a large number of laboratory tests and numerical simulation tests have been done by the research team. The results showed that the bearing capacity on the support was equivalent to that of U36 steel support, and it has a better yield ability to avoid stress concentration^31,32^.

**Figure 9:**
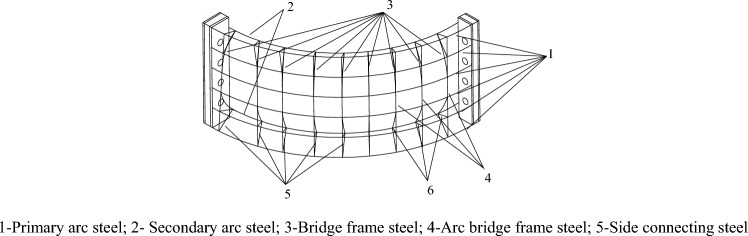
The monolithic shell of fully enclosed wire-shell support^33^.

The structure of each component is shown in Fig. 9. It can be seen from the figure that each member is composed of primary arc steel, secondary arc steel, bridge frame steel, arc bridge frame steel, and side connecting steel. It was found from the laboratory test that the primary and secondary arc steel bear the central pressure, while the rest of the steel mainly plays the role of connection. In addition, the steel wrapped the concrete, which can give full play to the advantage of concrete compressive capacity and weaken its tensile effect^31,32^. The connecting plate with bolt holes is welded at both ends of each component. Each support is composed of several component pieces and fastened with bolts. The support has a specific flexible pressure performance. In addition, the contractability plate can also be embedded in the joint to give the asset a certain degree of retractability. This kind of wire-shell support is installed one after another, and there is no interval between the supports. When the construction is completed, the wire-shell support supports many small-span bidirectional reinforced arch shells connected vertically and horizontally. It can entirely weaken the bending deformation of the reinforcement and enhance the three-dimensional bearing capacity of the support^33,34^.

Inspired by the invention principle 40 composite material principle, a kind of polypropylene fiber shotcrete was used in the roadway lining layer. According to the experimental laboratory test of our research group, the polypropylene fiber, a flexible fiber with low elastic modulus, can pull and draw the matrix around the crack when the shotcrete crack occurs, which can effectively improve the tensile resistance, bending resistance, and toughness of the shotcrete lining layer. Macroscopically, the failure model is a multi-seam fracture. Based on the test results, the optimal polypropylene fiber content was recommended at 0.9 kg/m^3^ ^35^.

Also, inspired by physical contradiction analysis, the technological process of assembly construction was adopted in the scheme of the fully enclosed wire-shell support structure. Specifically, the monolithic components were prefabricated on the ground and transported to the underground working face for assembly construction. This construction process reduces the intensity of underground work, simplifies the installation operation, improves the accuracy and quality of support construction, and achieves lightweight and continuous support.

### Advantages and application scope of the innovative scheme

This new support structure dramatically improves the support capacity of the traditional anchor net spray structure. In the early stage, adjustable support can be formed to complete the deformation of ground pressure. In the later stage, rigid support can be developed to withstand more significant ground pressure. In addition, the fully enclosed design effectively inhibits the deformation of the floor. Therefore, it belongs to a semi-rigid composite support structure. Thus, it is suitable for roadways under high ground pressure, large deformations surrounding rock, extremely broken soft rock, and dynamic pressure.

## Application of deep soft rock roadway repair engineering in Pansan Coal Mine, China

### Engineering situation

Pansan Coal Mine is located northwest of Huainan City, Anhui Province, China, 15 km north of Fengtai County, Huainan City. The length of the minefield is 9.6 km from east to west and 5.8 km from north to south, with an area of 54.3 km^2^. According to statistics, the rock roadway in Pansan Coal Mine has exceeded 50,000 m, most of which are soft rock roadways^36^. Due to the deeply buried, complex structure, high in-situ stress, and severe instability and deformation, the problem of soft rock roadway support has permanently restricted the safe and efficient production of Pansan Coal Mine.

Therefore, since the early stages of mine production, it has contacted scientific research institutions to research and construct soft rock roadway supports. The roadway support adopted are masonry arch, bolt-shotcrete, bolt-shotcrete-mesh, anchor injection, bolt-shotcrete-I bar, U-shaped steel support, the combined support of U-shaped steel support, and anchor cable mesh^37–39^. Although some promising results have been achieved, it has yet to fundamentally solve the problem of deep soft rock roadway support in coal mines in China.

**Figure 10:**
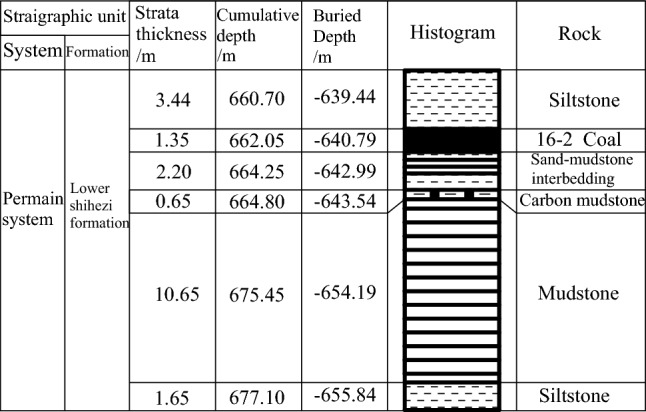
Locally drilled column of XII western 5 of Pansan Coal Mine.

The soft rock roadway is in the fourth-fifth section west of Pansan Coal Mine, with a buried depth of − 662 m; the surrounding rock of the roadway is mainly mudstone, and the rest is siltstone, carbon mudstone, and sand-mudstone interbedding. The borehole Fig. 10 is as follows. The roadway is located in the axial part of the syncline, the roadway deformation is severe, and the support is complex due to significant ground pressure. Due to the enormous pressure and severe roadway deformation, the 29 U-shaped steel supports were adopted before roadway repair. After repeated restoration, the loose circle of surrounding rock became larger and larger, and the U-shaped steel support was seriously damaged. The roadway broke down and had to be repaired frequently. Moreover, inadequate repair technology has made roadway deformation challenging to control. As a result, many workforce and material resources have been invested, and the maintenance costs remain high, which brings excellent hidden dangers to the mine safety production. According to geological and field investigation, it was determined that the repaired roadway is a typical large deformed soft rock roadway; moreover, after repeated maintenance, the loose circle was distributed, which resulted in deformation around the road, and the floor continued to develop. Therefore, it was decided to use the fully enclosed wire-shell support structure to repair the roadway.

### Design and processing of fully enclosed wire-shell support

According to the geological and damage conditions of the roadway in the fourth-fifth section of the west in Pansan Coal Mine, a fully enclosed wire-shell support suitable for the cross-section shape of the road was designed. The support width is 5300 mm, and the height is 4850 mm. The structure is shown in Figs. 8 and 9. The specific size and parameters are selected as follows.

**Figure 11:**
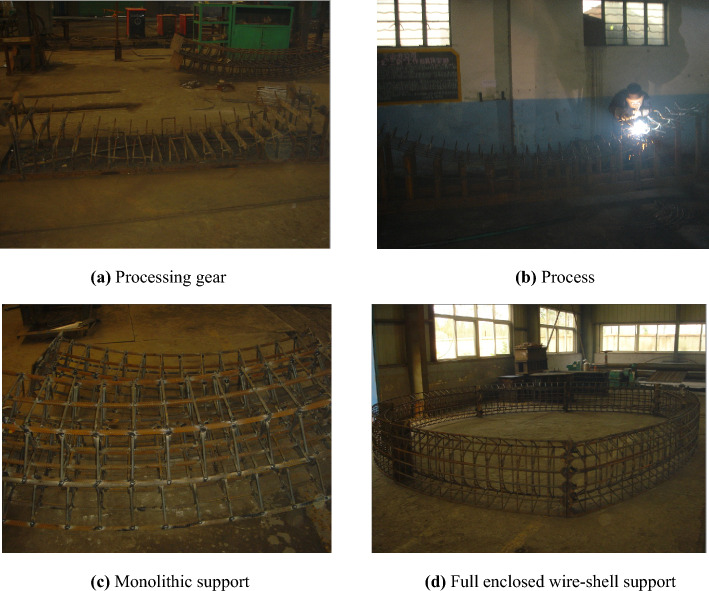
Monolithic shell and assembly of fully enclosed wire-shell support.

As shown in Fig. 8, a fully enclosed wire-shell support is divided into seven pieces according to the roadway section, which consists of one piece of roof shell, two pieces of upper side shell, two pieces of lower side shell, and one piece of floor shell. As seen in Fig. 9, each piece of shell is welded by two primary arc plates of steel of diameter 22 mm, six secondary arc plates of steel of diameter 20 mm, several bridge frame plates of steel, and arc bridge frame steels of diameter 12 mm. Several sides were connecting steels of diameter 12 mm. Each piece end of the shell is welded to a connecting plate with bolt holes. Assembling each piece shell, a contractibility plate with 20 mm is sandwiched between the two joints and then connected with bolts. The strength grade of shotcrete was C40, the mixing ratio was cement: medium fine sand: gravel: water = 1:1.16:1.90:0.38, and polypropylene fiber with 0.9 kg/m^3^ was mixed. The assembly effect of the single piece and complete, fully enclosed wire-shell support is shown in Fig. 11.

### Construction process

During construction, a piece of shell is prefabricated on the ground and then transported to the working face for assembly. The erection sequence of a fully enclosed wire-shell support is as follows: first two sides (including the upper side shell and the lower side shell), then the roof shell, and finally, the floor shell. The wire-shell supports are arranged individually, leaving no interval between them. The outer edge of each support must be in good contact with the surrounding rock. After the erection of one shell support, shotcrete should be filled immediately to ensure the quality of filling behind the support. If the interval is within 300 mm, it can be shotcrete directly. If the interval is too large, the interval can be filled with a wood wedge, gangue, or prefabricated concrete board.

In addition, to enhance the overall strength of the wire-shell support, one rock bolt with a downward movement of 45° is used at the lower part of each wire-shell support. Simultaneously, one rock bolt is installed above and below each side spandrel joint of the wire-shell support, which can support the wire-shell and induce the shear force and movement of the roadway shoulder. After 2 or 3 wire-shell supports are set up, it is necessary to check whether the section size meets the design requirements. After passing the inspection, C40 polypropylene fiber shotcrete can be poured. It is required that the shotcrete cover the wire-shell support and leave a protective of 10–20 mm thick. The floor wire shell was directly poured with C40 shotcrete.

### Monitoring analysis

**Figure 12:**
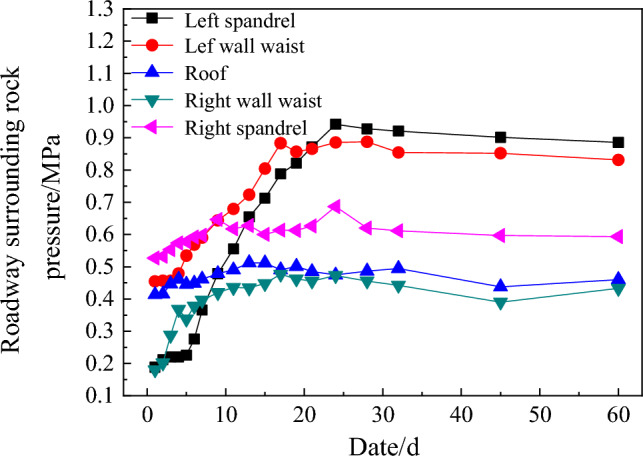
The curve of pressure and time of roadway surrounding rock.

**Figure 13:**
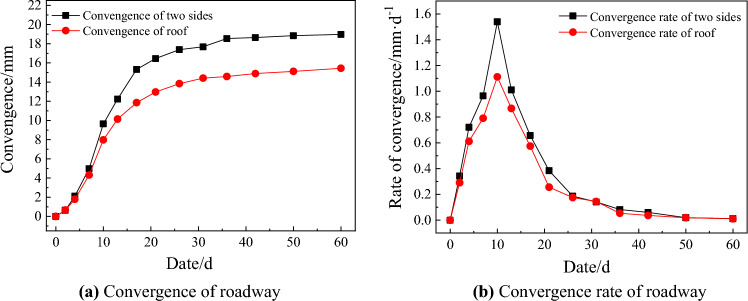
Convergence situation of roadway.

To study the stress and working performance of the enclosed wire-shell support structure. The monitoring content is divided into roadway surrounding rock pressure and roadway convergence deformation. The monitoring results are shown in Figs. 12 and 13. It can be seen from the field monitoring results that:

1. The roadway surrounding rock pressure of the fully enclosed wire-shell support structure increases gradually with time and tends to be stable after 30 days. It indicates that the wire-shell support structure has good deformation ability, and the surrounding rock deforms together with the support structure, which can release part of the surrounding rock pressure and improve the self-bearing capacity of the surrounding rock.
2. The convergence and deformation of the roadway surface are relatively less, and the intersection reaches a maximum of 20 mm after 60 days. Simultaneously, the deformation of the two sides and the roof is similar. It shows that the fully enclosed wire-shell support structure can effectively control the roadway deformation and make the surrounding rock appear to be a coordinated displacement, which realizes the stability control of the roadway. In the first ten days of support, the roadway deformation is large, and the convergence rate is relatively high, but its maximum is less than 1.5 mm/d. After 40 days, the convergence rate is only 0.05 mm/d, and the surrounding rock tends to be stable.

## Discussion and implications

This paper analyzed the problem of roadway support in coal mines from the TRIZ theory perspective, and the innovative scheme was put forward. From the standpoint of theoretical research, engineering practice, and economic effects, the implications of TRIZ theory are discussed as follows. Simultaneously, the limitations of this study are described.

For theoretical research, TRIZ theory believes objective regularities exist to solve problems. When people master these regularities, they can solve problems across fields and industries, which can consciously guide innovation, improve the efficiency of invention, and shorten the invention cycle. It is an excellent contribution of TRIZ theory to theoretical research.

For engineering practice, TRIZ theory can comprehensively analyze practical engineering problems, find the key points to solve problems, solve problems more fully, and develop new technologies more quickly. It is the functional significance of TRIZ theory to engineering practice. However, TRIZ theory must usually give conceptual ideas. For practical problems, the engineers and technicians must have professional knowledge in the field and combine it with the TRIZ theory, which can get a reasonable and effective solution to the practical problem. In this way, the transformation from idea to practice can be completed.

For economic and commercial impact, enterprises in China have begun to train their employees to master the TRIZ theory. It can effectively improve the efficiency of solving problems and create substantial economic and commercial value. However, there is still much room for the popularization and application of TRIZ theory.

For the study of this paper, at present, underground mechanization and unmanned are concerned and developed, and the new support structure of this paper to realize intelligent assembly is the future research direction. In addition, this study pays more attention to optimizing and improving the support structure, and the problem considered is relatively simple. In the practical application of roadway support engineering, geological conditions, construction environment, economic cost, and human factors all impact. A reasonable and feasible scheme should be formulated comprehensively considering many factors.

## Conclusions

Based on the works above, the following conclusions are drawn.

1. Based on the TRIZ theory, the system function and causal axis analysis of the roadway surrounding rock control were carried out to obtain the complex control problems of large deformation surrounding rock. The technical contradiction analysis, physical contradiction analysis, and substance and field model analysis were completed. The conceptual design of steel mesh shell support for soft rock roadway was proposed.
2. Inspired by the inventive principle of TRIZ, a fully enclosed wire-shell support structure technology was innovated. The installation speed is fast, which can provide continuous strong support to the roadway surface immediately after installation and effectively prevent the loosening of surrounding rock. Simultaneously, it also has a specific yield capacity. The fully enclosed inverted floor arch can effectively control the floor heave.
3. Combined with the typical roadway restoration engineering of Pansan Coal Mine, Anhui Province, China, the in-site construction and monitoring analysis were completed. The results show that this support can realize the co-deformation of surrounding rock and wire-shell support and effectively resist roadway large deformation. The maximum convergence was 20 mm, achieving a successful restoration of roadway support.
4. TRIZ theory is a highly effective and innovative method widely used to solve practical problems in various industries. It is a new attempt to reflect on the coal mine roadway surrounding rock control problems from TRIZ’s perspective, which extends the optimization design idea. This paper provides an excellent reference for a similar roadway support engineering design. It also has good reference value for other areas of the coal industry to use TRIZ theory to solve practical problems.
5. The issues of mechanization, artificial intelligence, underground worker safety, and efficient production have been widely concerned by society and enterprises in the field of roadway support to achieve less humanized, fast, efficient, and safe excavation support, which is the problem of this new support structure in the future development. In addition, there is still significant development in China to promote the use of TRIZ theory for engineering practice and improve the learning and training of engineering and technical personnel in enterprises.

## Data Availability

The datasets used and/or analyzed during the current study are available from the corresponding author upon reasonable request.
